# Interferometric Biosensor for High Sensitive Label-Free Recording of HiPS Cardiomyocytes Contraction *in Vitro*

**DOI:** 10.1021/acs.nanolett.3c04291

**Published:** 2024-05-22

**Authors:** Alessio Boschi, Giuseppina Iachetta, Salvatore Buonocore, Aliaksandr Hubarevich, Julien Hurtaud, Rosalia Moreddu, Marta d’Amora, Maria Blanco Formoso, Francesco Tantussi, Michele Dipalo, Francesco De Angelis

**Affiliations:** † Plasmon Nanotechnologies Unit, Istituto Italiano di Tecnologia, 16163 Genoa, Italy; ‡ Department of Bioengineering, University of Genoa, 16126 Genoa, Italy; § Department of Biology, University of Pisa, 56127 Pisa, Italy; ∥ Center for Research in Nanomaterials and Biomedicine, University of Vigo, 36310 Vigo, Spain

**Keywords:** Optical-cavity, cardiotoxicity, fluorescence, contraction, cardiomyocytes, in vitro

## Abstract

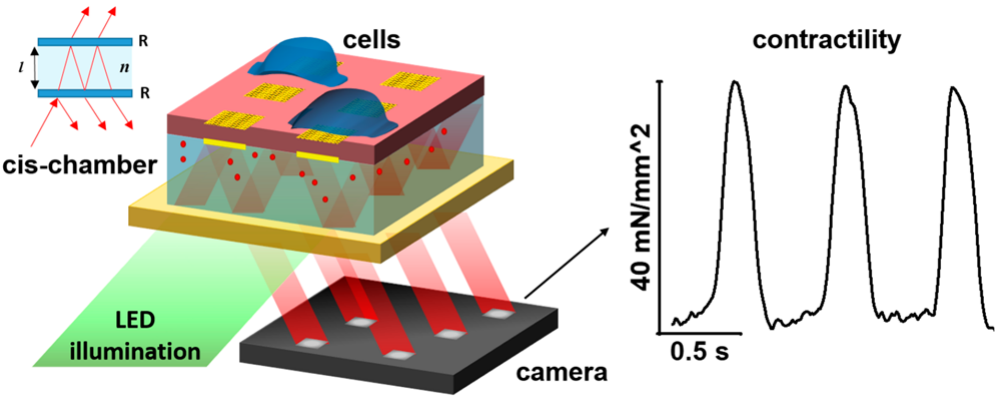

Heart disease remains a leading cause of global mortality, underscoring the need for advanced technologies to study cardiovascular diseases and develop effective treatments. We introduce an innovative interferometric biosensor for high-sensitivity and label-free recording of human induced pluripotent stem cell (hiPSC) cardiomyocyte contraction *in vitro*. Using an optical cavity, our device captures interference patterns caused by the contraction-induced displacement of a thin flexible membrane. First, we demonstrate the capability to quantify spontaneous contractions and discriminate between contraction and relaxation phases. We calculate a contraction-induced vertical membrane displacement close to 40 nm, which implies a traction stress of 34 ± 4 mN/mm^2^. Finally, we investigate the effects of a drug compound on contractility amplitude, revealing a significant reduction in contractile forces. The label-free and high-throughput nature of our biosensor may enhance drug screening processes and drug development for cardiac treatments. Our interferometric biosensor offers a novel approach for noninvasive and real-time assessment of cardiomyocyte contraction.

Contractility dysfunction of the cardiac tissue is one of the major causes of heart disease, formally known as cardiovascular disease (CVD) which accounts for 31% of all global deaths.^1^ Micro- and nanoengineered biosensors are a suitable technology to investigate cardiomyocytes (CMs) contractility in physiological and pathological conditions.^2^

Human induced pluripotent stem cell cardiomyocytes (hiPSC-CMs) is acknowledged as the best candidate to model the fundamental properties of the cardiac muscle.^3^ HiPSC-CMs maintain the ability to contract spontaneously.^4^ Many *in vitro* platforms have been developed with the aim of monitoring and quantifying the contraction force of hiPSC-CMs monolayer.^5−11^ The contraction is evaluated by measuring the following parameters: contractile force, the dynamic of contraction, beating rate, passive tension, synchronicity, and beating propagation. Detecting alterations in any of these parameters can discriminate potential indications of disease in patients or toxicity during a drug assessment.^12^

Gold standards to measure CMs contractility are atomic force microscopy (AFM) and traction force microscopy (TFM).^13,14^ AFM only allows measurement of single-cell contractility while TFM requires the insertion of markers in the cell environments.^13,15,16^ Force measurements on CMs monolayers involve electrical impedance-based sensors,^5,7^ image-tracking-based sensors,^17^ and thin-film-based sensors.^18^ Such distinction does not preclude these approaches to be combined toward the design of a low invasive, high throughput, and user-friendly heart-on-a-chip platform.^19^ The first two technologies suffer from invasiveness^14,20−22^ and difficult data interpretations.^7^ The thin-film-based sensors stand out as the most promising technique for contractility assessment in terms of invasiveness, throughput, and easy manufacturing.^18,20,23^ In such sensors, CMs monolayer is cultured on a thin polymeric membrane.^8,9^ The CMs beating induces a membrane displacement which is measured by capacitive-, resistive-, or optical-based technique and converted into a force.^9,10,24,25^ Currently, such devices do not allow studying the contribution of single cells to the monolayer contraction across the culture.^26^ Among thin film-based biosensors, optical-based measurements remain the most promising approach to achieve label-free, high throughput, and accurate measures of the contractility of CMs.^27^

In this work, we present a new optical thin-film-based device concept. The device is designed as an array of optical interferometers that enable label-free high-resolution imaging of spontaneous and paced hiPSC cardiomyocyte contraction *in vitro*. The device, schematically depicted in Figure 1, is an array of *microinterferometers* placed underneath the cell culture. By monitoring the *microinterferometers* modulations, one can monitor the cell beating. Thanks to the interferometric technology, our device allows multipoint monitoring of the cardiac-induced substrate displacements down to tens of nm; it is thus compatible with confocal applications and calcium imaging (Supporting Information Section 3). We report the device architecture, a set of experimental data, and numerical calculations on both mechanical and optical behavior of the device. We calculated a contractility traction stress of 34 ± 4 mN/mm^2^. This traction stress value is compatible with measurements performed on similar cardiac cellular models using well-established techniques (https://innovitro.de/wp-content/uploads/2021/10/210911-innoVitro-Contractile-Force-Flyer.pdf).

**Figure 1:**
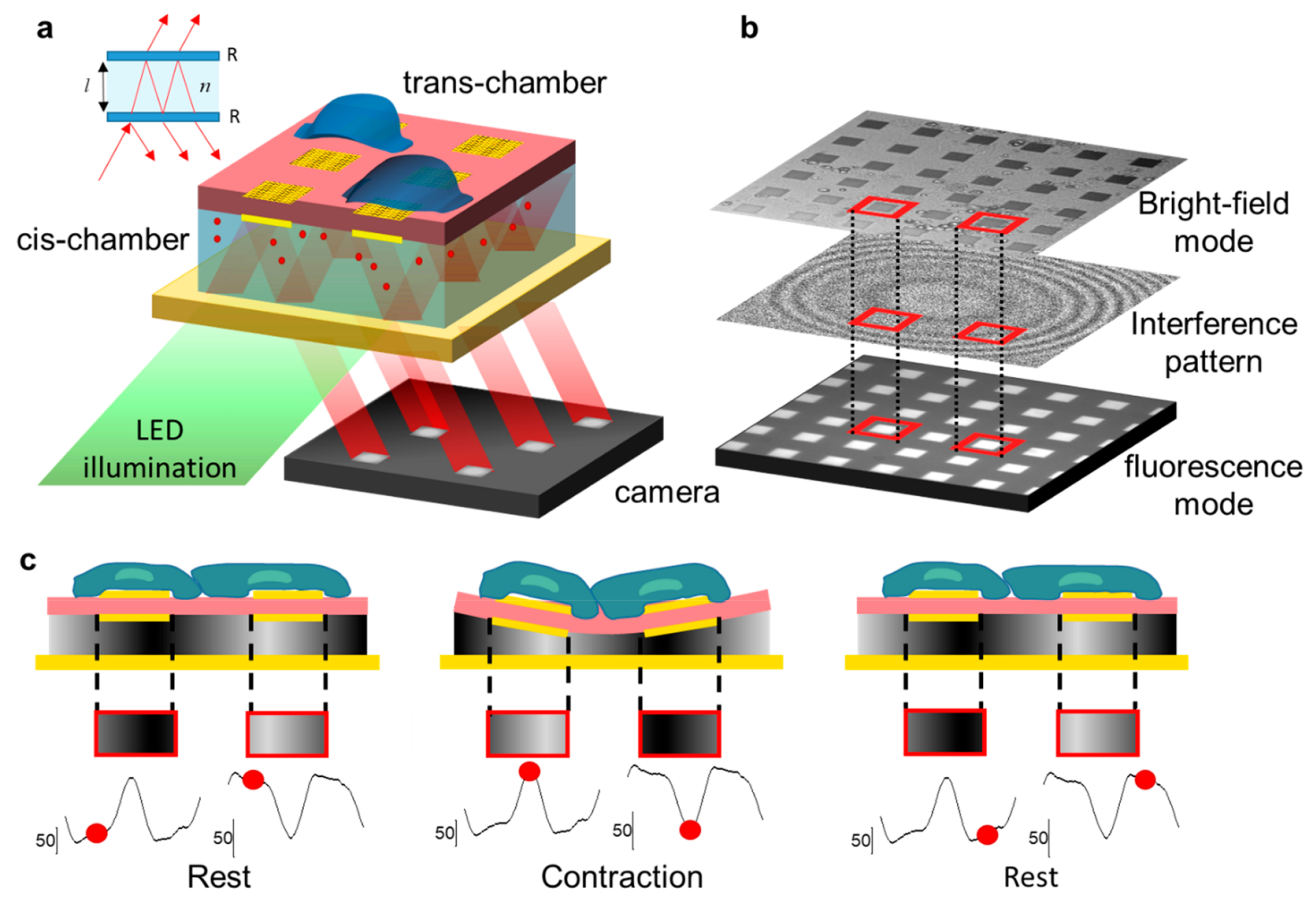
Device working principle. (a) 3D scheme of the device representing the silicon nitride membrane (red) with the embedded matrix of gold mirrors. On top of it, in the trans-chamber, the cell environment contains a 2D monolayer of hiPSC-CMs (shown only two exemplary cells). The cell monolayer is attached to the silicon nitride membrane and the porous gold squared pads. The cis-chamber under the membrane is filled with ethylene glycol where we dissolved 0.2 mg/mL rhodamine (red dots). When the chamber is irradiated by the tailored light beam, the fluorescence in the cis-chamber is enhanced. The array of square gold mirrors reflects the fluorescence signal that is acquired by a CMOS camera. (b) Schematic overview of the signal acquired by the camera: first, in bright field mode it is possible to image the cell culture, second the interference pattern created in the cis-chamber, third the down-sampled interference pattern reflected to the camera sensors. (c) Phase shift of the optical pattern and the relative fluorescent signal reflected by two single gold pads during the contraction event. During the contraction, the cells exert a force that bends the membrane, obtaining a variation in the cavity size. The variation of the cavity determines a change in the light intensity acquired by the camera (scale bar: photocounts).

The device technology, described in Figure 1, is based on the concept of “optical cavity” in classic Optics: an optical cavity is an arrangement of mirrors or other optical elements that form a cavity resonator for light waves.^28^ Inside the resonator, interference phenomenon occurs, creating a pattern that can be modified consistently with the parameter of the optical cavity (e.g., the distance between the two mirrors).^29,30^ Following this idea, we designed a device consisting of two optical interfaces. Cardiac cells are cultured on the upper and oscillating interface that is made of a thin silicon nitride membrane patterned with squared gold mirrors on both sides (Figure 1, Figure S5). At the interface with cells, squared pads of nanoporous gold enhance the cell’s adhesion to the membrane,^31^ while most of the area remains transparent allowing for easy cell manipulation and observation with an inverted microscope setup as well as optical methods for electrophysiological applications such as calcium imaging (Supporting Information, Figure S5). The upper interface also delimitates and encloses the cell environment, namely, the trans-chamber. The second and fixed interface, placed below the cavity, is a square glass substrate coated with a thin layer of gold. The cavity, namely the cis-chamber, defining space between the two interfaces, is filled with a fluorescent molecule (rhodamine, R6G, 0.2 mg/mL) dissolved in a nonvolatile liquid (ethylene-glycol). As shown in Figure 1a, when irradiated by the correct wavelength light enhancing fluorescence emission of R6G (λ_ex_ = 543 nm), an interference pattern is created between the two interfaces. The fluorescence signal belonging to the volume under each gold mirror is reflected in the camera detector, as shown in Figure 1b. While the cardiac cells are resting the optical signal is steady. During the contraction phase, shown in Figure 1c, cardiac cells apply longitudinal forces to the silicon nitride membrane that lead to a local change of the distance between the two faces of the optical cavity. Hence, during cell contraction, each gold mirror behaves as the oscillating part of an interferometer, in which the distance *l* changes (inset Figure 1a, Supporting Information: eq 1). As a result, the optical signal derived from each *microinterferometer* changes according to the cells contraction (see Section 3.4). In this perspective, the device allows for monitoring an entire cell culture contraction by using a matrix of *microinterferometers* which changes its parameters according to the cells activity. Notably, the device configuration can be easily tuned by modifying the fabrication parameters of the *microinterferometers,* such as the silicon nitride membrane thickness,^32^ hence the substrate stiffness, the thickness of the metallic coatings of the mirrors,^33^ and the media filling the cavity.^34^

To demonstrate the capability of our device, we used a hiPSC-CMs. The biocompatibility of the substrate was previously tested in a separate study,^35−38^ confirming cell viability of approximately 90% after 7 days *in vitro* (DIVs). After 13 DIVs, the sample with mature cells is assembled on the microscope stage for measurements. A drop of fluorescent solution (0.2 mg/mL, R6G in ethylene-glycol) is placed on the bottom mirror, and the silicon nitride membrane with the cells is positioned above it, enclosing the liquid between the two surfaces. During the measurement, the sample is illuminated with a wavelength of λ_ex_ = 543 nm to enhance fluorescence emission from R6G molecules (Figure 2e). When irradiated from the bottom, each *microinterferometer* signal in the camera field of view is acquired simultaneously at a frame rate between 50 and 1000 fps. Each recording lasted between 30 and 120 s for a total time of up to 30 min per experiment. Figure 2a shows the fluorescence signal intensity from a single *microinterferometer*.

**Figure 2:**
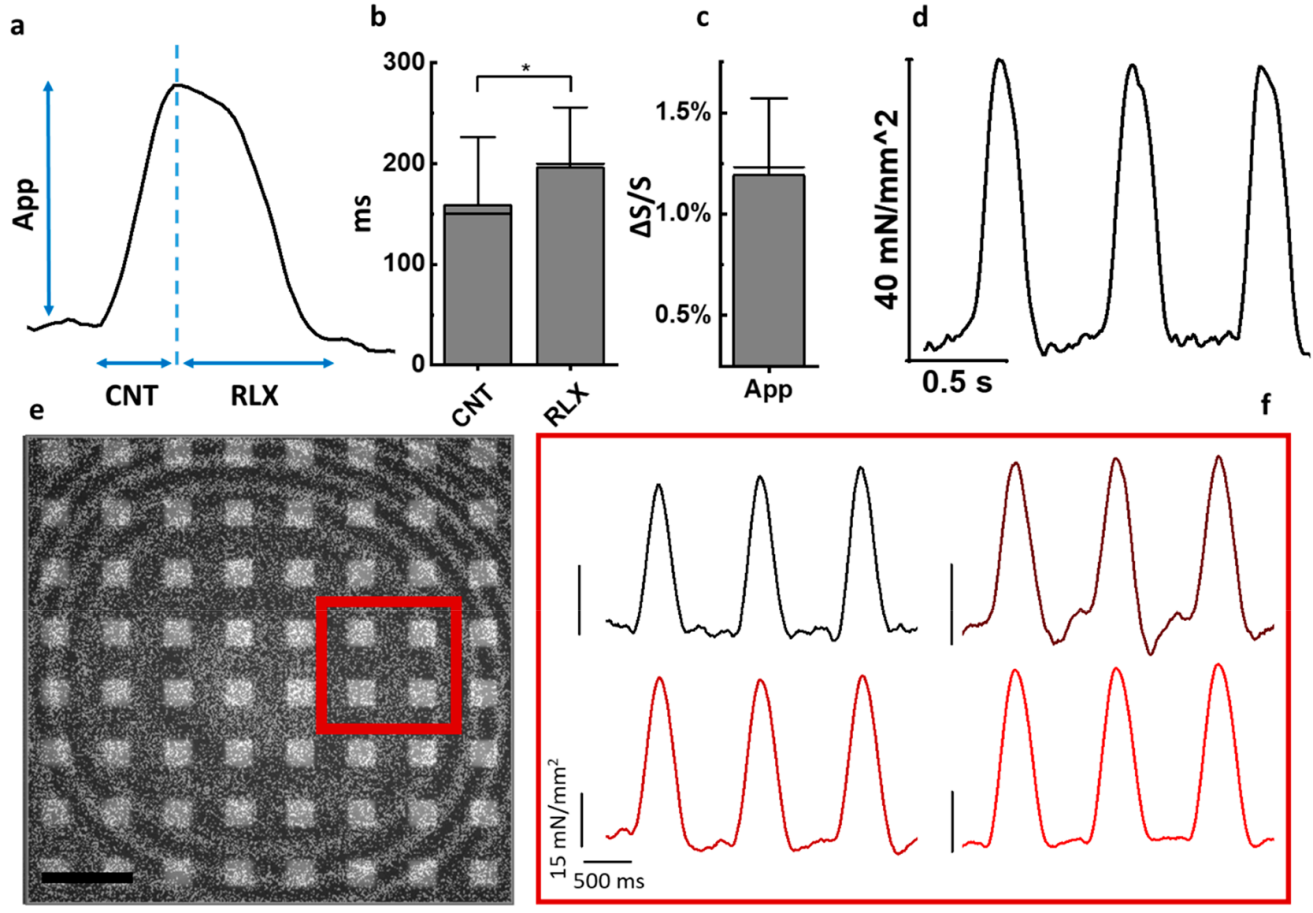
Recording of contraction activity of hiPSC-CMs (FUJIFILM Cellular Dynamics, Inc. (FCDI)) (a) Trace of a single contraction event. (b) Bar plot that quantifies the difference between contraction time (CNT) and relaxation time (RLX) (Kruskal–Wallis, *p* < 0.01). (c) Bar plot to quantify the signal-to-noise ratio of the fluorescence signal defined as Δ*S*/*S*. (d) Example trace of 3 contraction events from a single *microinterferometer.* (e) Fluorescence image of the device with superimposed the interference pattern (scale bar: 90 μm). (f) Four contractility traces from the same recording showing the variability of the signal across an area of the device (*x* scale bar: 0.5 s; *y* scale bar: 15 mN/mm^2^).

During cell contraction, the membrane oscillations cause changes in the fringes of the interference pattern (Figure 2e), thus causing variations in fluorescence intensity in each single pad. By monitoring the periodic variation of fluorescence intensity, we monitor the periodic cell beating. In other words, by monitoring the movement of the fringes, i.e., the signal of a *microinterferometer*, we measure the variation of the spatial phase of the interference pattern (see video 1). If the size of the interference bands is roughly equal to the size of each *microinterferometer* (30 μm), the horizontal movement of the bands will result in the *microinterferometers* switching from a dark to a bright band or from a bright to a dark band, thus maximizing the sensitivity (Supporting Information Figure S2).

The frequency of the signal depends on the spontaneous beating rate of the cell culture,^39^ which is approximatevly 1 Hz at 37 °C. Figure 2a provides details of a single contraction event, demonstrating the device’s ability to discriminate between contraction and relaxation. The signal-to-noise ratio (SNR), computed as the ratio between the fluorescence peak-to-peak signal of a contraction event (A_pp_) and the noise intensity when the cell is at rest, is approximately 10. Moreover, as described in detail in section 3.4, knowing the mechanical properties of the membrane and the optical properties of the cis-chamber, we can quantify the contractility strength of the cardiac cells. Figure 2d shows three contraction events recorded from a single gold sensor that measured a peak contractility of 38 mN/mm^2^. Across the whole device, the amplitude and shape of the contractility signal can slightly differ according to the location of the single sensor (Supporting Information Section 2). In Figure 2e, we report four traces with matched interference patterns and interferometer size providing a contractility of 34 ± 4 mN/mm^2^.

To further assess the capabilities and versatility of our device, pacing experiments are conducted by applying a voltage stimulus to induce controlled contraction frequencies (Figure 3a–b). Paced experiments provide a controlled and reproducible platform for investigating the mechanisms underlying cardiac arrhythmias and other heart-related disorders.^40^ Thereafter, we explore the effects of Blebbistatin on the contractility amplitude over time following its administration (Figure 3c–e). Blebbistatin is a molecule that decouples the mechanical contraction of cardiomyocytes from their electrophysiological activity.^41^ The main effect of Blebbistatin is to reduce or stop the mechanical contraction while maintaining the electrical activity.^42^

**Figure 3:**
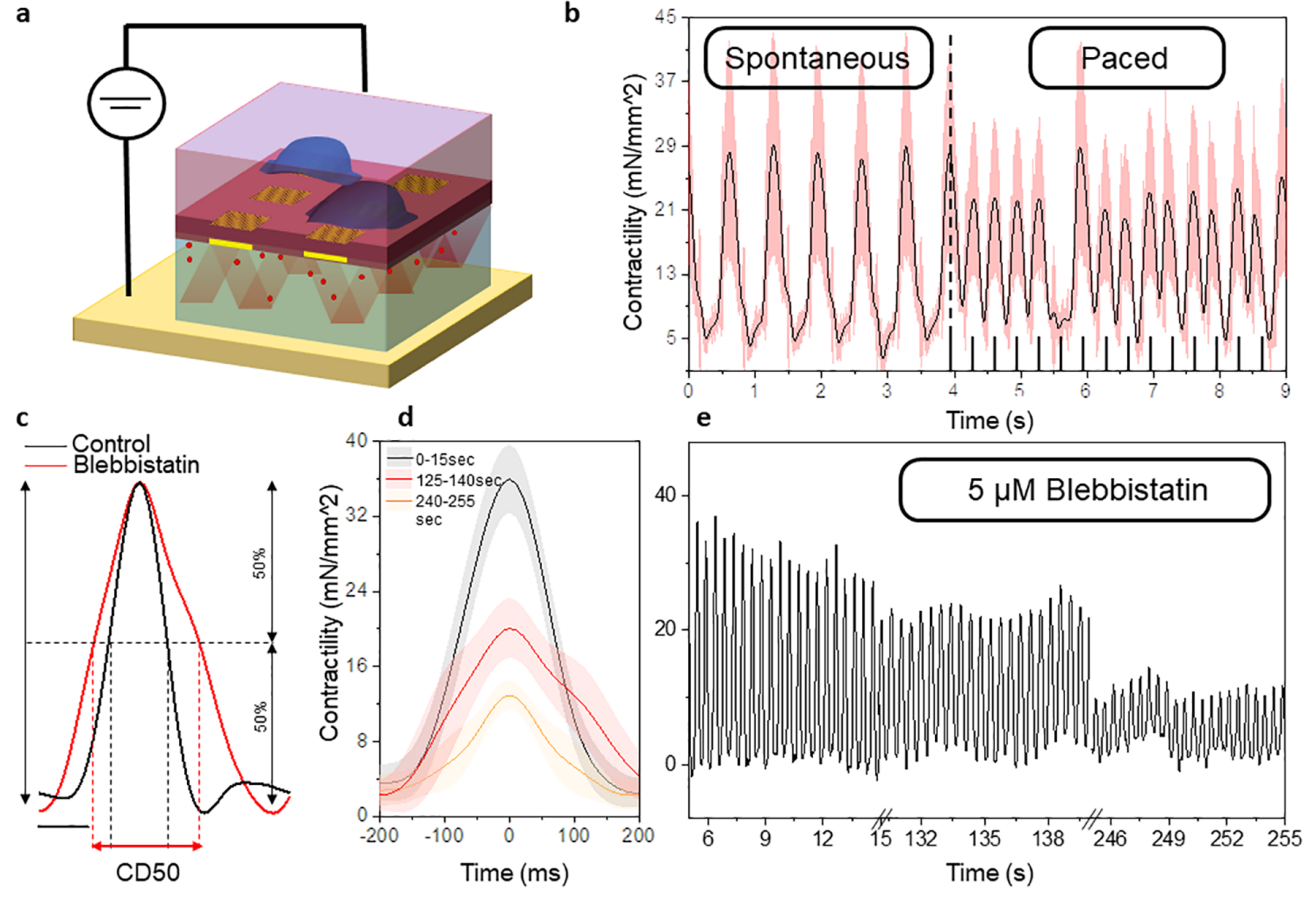
Paced and drug detection measures. (a) Scheme the device during paced stimulus configuration. A voltage difference is applied across the cis- and trans-chamber (b) 10 s of contractility measure over 6 gold *microinterferometer* during pacing stimulation (mean ± standard deviation). (c) Normalized averaged contraction traces in drug assessment condition. The black line represents the trace in the control condition, and the red line represents the contractility trace 2 min after the BB administration. (d) Contractility amplitude for different time intervals after the BB administration. (e) Mean contractility recording for 260 s after BB administration.

In the pacing experiments, we apply a voltage-pulsed stimulus between the cis- and trans-chambers (Figure 3a). After recording a baseline of spontaneous activity, we stimulate the cell culture, achieving a paced contraction frequency of 3 Hz with a voltage of 2 V and a pulse duration of 200 μs (Figure 3b). Subsequently, we administered 5 μM of Blebbistatin to the cell culture and promptly commenced monitoring cell spontaneous contractility (Figure 3e). After 2 min, a significant 30% decrease in the contractility amplitude is observed, that reached nearly 70% after 5 min (Figure 3d). Moreover, our device is capable of detecting relevant changes in the contraction profile during drug assessment: Figure 3c displays the averaged contraction shape over a 20 s activity before and after the administration of 5 μM of Blebbistatin. The contraction duration at 50% of the peak amplitude (CD50) is shown in Figure 3c, confirming previous results in literature.^43^ The CD50 before the administration was 159 ± 47 ms, while after administration, it increased to 213 ± 51 ms.

To gain insights into the origin of the optical phenomena observed in our device and quantify the contraction forces applied by the cells to the membrane, we have shown simulations to study the behavior of the electromagnetic field in the cis-chamber. Figure 4b presents a cross-sectional schematic of the simulation setup. A 2D model of the cis-chamber reflects the actual sizes and materials of the device. The model consists of a silicon nitride membrane (*L* = 500 nm, *n* = 2.02) covered by a gold layer (H2 = 200 nm). The membrane was separated from the bottom glass (G2 = 500 μm, *n* = 1.45) by a layer of ethylene glycol (G1 = 15 μm thick, *n* = 1.43). Additionally, the bottom glass is coated with a thin gold layer (H1 = 20 nm). For the simulation, a dipole source with a wavelength of 592 nm represented the irradiation light, and a virtual monitor was placed at a distance of G3 = 0.5 mm from the bottom glass.

**Figure 4:**
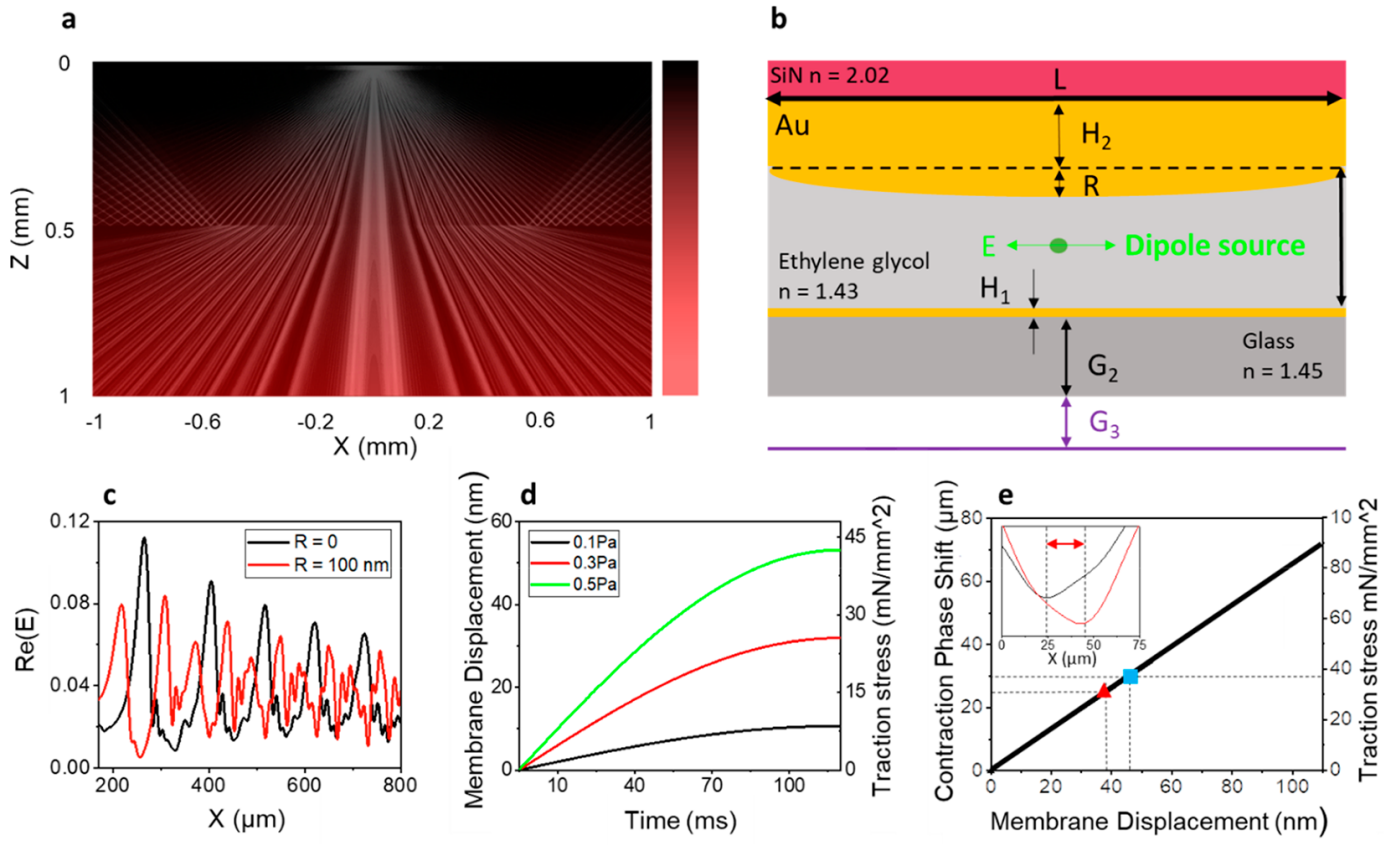
Characterization of optical cavity and membrane mechanical properties. (a) 2D simulation of the electromagnetic field generated by a dipole source in the cis-chamber. (b) Scheme of the simulated cavity. (c) Line profile of the simulated interference pattern for different values of R. The spatial difference between the negative peaks quantifies the contraction phase shift. (d) Mechanical characterization of the silicon nitride membrane. We simulated the membrane displacement under different values of normal load pressure (Pa) and we computed the relative traction stress along the longitudinal direction (mN/mm^2^). The normal load was applied as a 2 Hz sinusoidal pressure with maximum values of 0.1, 0.3, and 0.5 Pa. (e) Shows the linear relationship between the simulated interference phase shift, the simulated membrane displacement, and the simulated traction stress during the bending. The blue square highlights the 30 μm phase shift that corresponds to the size of the *microinterferometers.* The red triangle highlights the experimental phase shift observed on the real device during cardiomyocytes contraction.

We present two different simulations. First, we consider the device in a static condition, where the two surfaces of the cavity are parallel. Second, we simulate the scenario in which the membrane bent under the contraction forces applied by the cells, causing the two surfaces to no longer remain parallel. To simulate the bending effect, we introduced the parameter *R*, representing the curvature of the membrane at different levels (*R* = [50, 100, 150 nm]). The first condition confirms that it is possible to obtain optical interference in the cis-chamber that closely matches the experimental observations, crucial insights into the underlying physics of our device (Figure 4a). In the second condition, we explore the impact of membrane bending and its influence on the interference patterns (Figure 4b–c). The simulations reveal that as the bending parameter R increases, the phase of the optical interference shifts accordingly (Figure 4c). Consequently, the interference patterns captured by the virtual monitor exhibited variations that correlate with the extent of membrane deflection due to the applied contraction.

Afterward, we investigated the dynamic behavior of the silicon nitride membrane to understand the forces that could cause it to bend within the range of displacement observed in the optical phase shift. We present finite element analysis (FEA) in COMSOL Multiphysics. The deformation and stress fields are analyzed across the membrane for different loading pressure scenarios (Supporting Information Section 1). The simulations are performed by varying the amplitude (0.1, 0.3, and 0.5 Pa) of a quasi-static (2 Hz) pressure load applied on the top membrane face. Figure 4d shows the temporal relationship between the applied quasi-static pressure load and the resulting maximum vertical membrane displacement (*y*-axis on the left) and longitudinal traction stress across the membrane (*y*-axis on the right).

In Figure 4e, we present a comprehensive graph that establishes a correlation between numerical simulation data and the experimental results. The graph showcases the simulation outcomes, demonstrating the shift and the average traction stress experienced during the deformation process (indicated by the black line). The inset shows an example of experimental phase shift during the contraction event that we quantify as 25 ± 5 μm. The calculation of the phase shift, both in the simulated and experimental data sets, involved determining the spatial difference (μm) between the negative peak values of the interference line profile. The red triangle on the graph indicates the actual experimental phase shift data point, effectively placed along the characteristic line predicted by the simulations. Furthermore, the blue square indicates the anticipated membrane displacement and, consequently, the expected traction stress, corresponding to a phase shift of 30 μm, which aligns with the actual size of our gold *microinterferometers*.

The results presented in this study demonstrate the successful development and application of an interferometric biosensor for the highly sensitive and label-free recording of hiPSC cardiomyocyte contraction *in vitro*. The device is able to capture CMs beating frequency and the contractile forces exerted by the CMs monolayer. We quantified an averaged contractility of 34 ± 4 mN/mm^2^ at 13 DIVs, in agreement with the state-of-art literature (https://innovitro.de/wp-content/uploads/2021/10/210911-innoVitro-Contractile-Force-Flyer.pdf). The device is capable of accurately detecting and quantifying the forces applied during paced contractions and, notably, successfully discriminating variations of contractility during drug assessment.

The key point enabling these results is the design of an optical cavity whose parameters are modified by CMs contraction. The technology detects displacements on the order of tens of nanometers. This accuracy in displacement measurement allows thin silicon-based membranes to be adopted as flexible substrates, despite its high Young’s modulus.^40,44^ Silicon-based materials are gold standard in micro- and nanofabrication, which represents an advantage to scale up the fabrication process toward high throughput.^45^ Polymeric-based devices cannot simultaneously image the cell culture: the displacement of the polymeric membrane during contraction is in the order of 10 μm,^46^ that is twice the depth of field of a standard 20× objective. In contrast, since the nanometric range displacement of the silicon nitride membrane does not significantly affect the optical focus of the objective, our biosensor enables the simultaneous measurement of contraction and imaging of the cell culture. The *microinterferometer* configuration allows one to observe the cell culture at the inverted microscope, ensuring compatibility with optical methods for electrophysiological applications such as calcium imaging and confocal imaging (see Supporting Information Section 3).

Our perspective pushes the state of the art since is a first step toward a multipoint acquisition of the monolayer contractility. The silicon-based *microinterferometers* are the same size as the CMs and the spatial resolution of the device could reach *single-cell* resolution in optimized configurations. Although in our device the contractility still remains a measure across the whole CMs monolayer, future improvements can lead to a single-cell resolution contractility measure of the CMs monolayer. We exploit 900 *microinterferometers* distributed on the 2 × 2 mm^2^ membrane to ensure that the period of the interference pattern in a subarea of the device would fit the *microinterferometer* size, enabling the reliable detection of the CMs contraction. The location of the optimal working area can change among different devices because the perfect alignment of the cavity interfaces is practically trivial (Supporting Information Section 2). Therefore, controlling the origin of the interference pattern and thus the area of optimal working remains a challenge. In spite of this, we always obtain a considerable number of *microinterferometers* (in the order of 50) working in the area where the period of the interference matches the *microinterferometers* size, and the sensitivity is maximized (*n* = 9). The signal coming from *microinterferometers* in the area where the sensitivity is maximized is averaged to obtain a robust measure of the membrane displacement.

A limitation of the proposed approach is that flexible and extracellular matrix-like substrates are usually considered to better mimic physiological mechanical stimulus in the cell environment.^47,48^ Notwithstanding, flexible materials, such as PDMS, combined with silicon-based substrates and the proper fabrication processes can lead to a new family of devices able to provide the proper mechanical stimulus and properties for both cell development and high-resolution contractility measures. Toward this goal, parameters of the *microinterferometers*, such as size, reflectivity, and refractive index, can be optimized and modified. In detail, different excitation wavelengths, solvents, and fluorescent molecules can be explored to obtain interferences suitable for single-cell detection.

In this study, we introduce an innovative technology for the *in vitro* measurement of contractility in CMs monolayers by taking advantage of the microinterferometric approach. Through a set of experiments and numerical simulation, we show the capability to assess label-free contractility of hiPSC-CMs monolayers. Notably, the device can estimate various important parameters such as frequency, shape, and amplitude of the contractile force generated by the cell culture. We have also demonstrated that the device can be interchangeably used for calcium imaging, enabling multimodal characterizations of cardiac cells. Although the current architecture operates at the level of whole cell culture, the concept can be translated to smaller scale by proper device design to reach single cell resolution. The latter will help to distinguish the diverse contributions of individual cells to the overall tissue contraction and most importantly to study propagation patterns and reentry occurrences. Also, the concept can be extended to various scenarios in which biological tissues exert forces that can be translated into measurable displacements.

## Supporting Information Available

Additional experimental details, materials, methods (PDF)
Video acquisition 1 (AVI)
Video acquisition 2 (AVI)
Video acquisition 3 (AVI)

## Supplementary Material

nl3c04291_si_001.pdf

nl3c04291_si_002.avi

nl3c04291_si_003.avi

nl3c04291_si_004.avi
