# Supplementary material for: Interferometric Biosensor for High Sensitive Label-Free Recording of HiPS Cardiomyocytes Contraction in Vitro
Source: Nano Lett. 2024 May 22;24(22):6451–8. doi: 10.1021/acs.nanolett.3c04291 (PMC11157657; doi:10.1021/acs.nanolett.3c04291)
Supplement: Supplementary file 1 — nl3c04291_si_001.pdf [file nl3c04291_si_001.pdf]

Support Information

Interferometric biosensor for high sensitive label-free recording of HiPS cardiomyocytes contraction in-vitro

Alessio Boschi[1,2], Giuseppina Iachetta[1], Salvatore Buonocore[1], Aliaksandr Hubarevich[1], Julien Hurtaud[1], Rosalia Moreddu[1], Marta D'Amora[1,3], Maria Blanco Formoso [1,4], Francesco Tantussi [1], Michele Dipalo *[1], Francesco De Angelis *[1]

* Corresponding authors: francesco.deangelis@iit.it, michele.dipalo@iit.it
[1] Plasmon Nanotechnologies Unit, Istituto Italiano di Tecnologia, 16163 Genoa, Italy
[2] Department of Bioengineering, University of Genoa, 16126 Genoa, Italy
[3] Department of Biology, University of Pisa, 56127 Pisa, Italy

1 Mechanical Simulation

The predictions of the numerical simulations arevalidated by using the analytical results available in literature. The membrane considered in this study, due to its aspect ratio (L = 2mm, thickness = 500 nm) and elastic properties (E= 250 GPa) can be approximated as a thin plate[1] which dynamic has an upper bound in the membrane-like model and a lower-bound in the thick-plate model (the transition between the two dynamic regimes is dictated by the non-dimensional parameter $\frac{p}{E}\left(\frac{a}{t}\right)^4$). We verified the validity of both the models which admit closed form solutions under an uniform load[2,3]. We therefore verified the match between the analytical and numerical predictions for both these models within the respective range of applicability. As result, we finally validated the accuracy of the numerical results in the range of material, geometric and pressure parameters of interest for this study, which is intermediate between the two models introduced above but for which, to the best of our knowledge, no closed form solutions are available [4]. We investigated numerically applied pressure loadings of amplitude 0.1, 0.3 and 0.5 Pa determining the corresponding maximum displacements and traction stresses that are reported in Figure 3d. The model of the thin plate implies stresses only on the plane of the surface and those stresses are uniform throughout the thickness of the membrane[5]. Due to the smallness of the deformations investigated (with respect to the characteristic dimensions) the membrane behavior is expected to obey the linear elastic regime. The numerical simulations of membrane mechanical deformations show a linear relationship between vertical displacement and traction stress. It predicts, for a quasi-static applied load 0.5Pa, a traction stress and vertical displacement of 40mN/mm^2 and 50nm. These values confirm the validity of the linear elastic model, is the maximum stress is well below the yield stress of silicon nitride, typically within the range 60 -520 MPa.

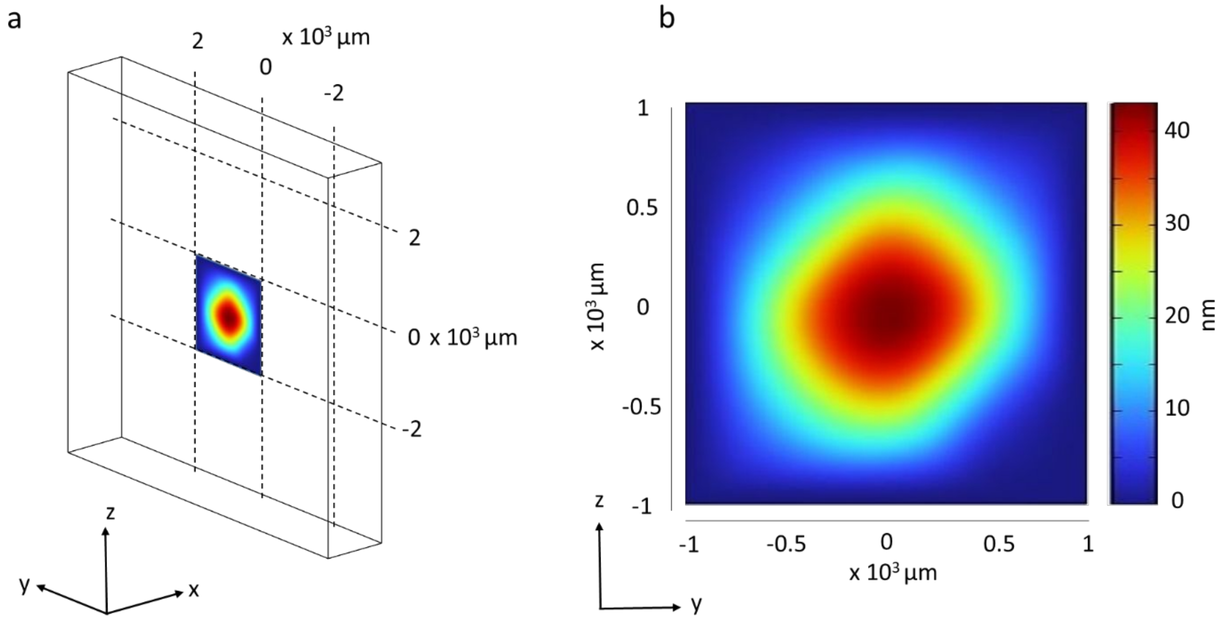

Figure S1: a) Geometry of the thin plate modeled in COMSOL. b) Displacement simulation of the thin plate model under a vertical load equal to 0.5Pa.

2 Identifying Areas of Maximum Sensitivity

Among the optical read-out methods, our approach stands out as the first interferometric based biosensor designed to measure and quantify the forces applied during the contraction of a 2D monolayer cardio myocyte culture. Briefly, interferometric biosensors measure small variations occurring in an optical beam during his pathway. When the optical path of a light wave λ is confined between two interfaces separated by a distance *l*, in a media with refractive index *n*, his phase φ can change following Equation 1:

$$\varphi = \frac{2\pi\, l\, n}{\lambda}$$

Equation 1

Although changes in the refractive index are commonly used to detect variations in the phase, small changes in the distance *l* between the two mirrors can drive the cavity off resonance and phase-shift light out of the interferometer, leading to considerable alterations in the beam characteristic [6]. In our device, the distance *l*, the distance between the two interfaces, can change under the longitudinal forces applied by CMs contraction. The intrinsic properties of the *micro-interferometer* sensor allow the device to have a suitable dynamic range at the *nm* scale.

In this study, we fabricated and successfully measured 9 devices. Although we managed to create the interference pattern across the different devices, its precise location and shape slightly changed. Such minor variations can be attributed to several reasons. Firstly, the devices were fabricated from commercial wafers (Silicon Materials), which exhibit a variability of ±25nm in the thickness of the silicon nitride layer. Secondly, the membrane itself can bend in different modes when in contact with a fluid (ethylene glycol). Lastly, the local forces of cardiomyocytes and their distribution across the membrane differ in various

cultures, even though the plating parameters remain consistent. While the aforementioned phenomena are beyond our control, the strategy of patterning the entire device surface and creating a matrix of micro-interferometers ensures that the device can detect the signal wherever the interference pattern is effectively generated. During measurements, we recorded different areas across the device separately, using acquisition sets lasting 1-2 minutes each, resulting in a total recording time of approximately 30 minutes. In the post-processing of the data, we identified the areas of maximum sensitivity of the device, as explained in Figure S2. These areas of maximum sensitivity align with the size of the interference pattern, and the identification process was carried out as follows: First, we employed ImageJ software for background subtraction, subsequently, we used the Tox-Free software to automatically identify the region of interest and extract the relative fluorescence intensity traces. The final measure of contractility is averaged across the micro-interferometers displaying maximum sensitivity. In Figure S3, we provide an example of the post-processing steps required to identify the areas of the device from which to extract the contractility signal. Initially, we opened the video in ImageJ and subtracted the first frame from the video to eliminate the absolute value of the light intensity across the images (Figure S3a). In this configuration, the interference phenomenon became clear, and we identified the pads with the potential for higher sensitivity (Figure S3b, marked with green squares). We selected the same pads in the custom Tox-Free software, where we extracted the fluorescence traces (Figure S3c). This example (Figure S3c) shows the signal derived from the four selected pads with a clear signal-to-noise ratio (S/N). Averaging the signal from these four pads provided a more precise measure of the contraction activity. Subsequently, in Figure S4, we presented measurements from another set of four pads within the same recording. These additional pads were located in an area of the device where the interference fringes were smaller than the micro-interferometers (approximately half the size). In this area (Figure S4c), the S/N was notably lower.

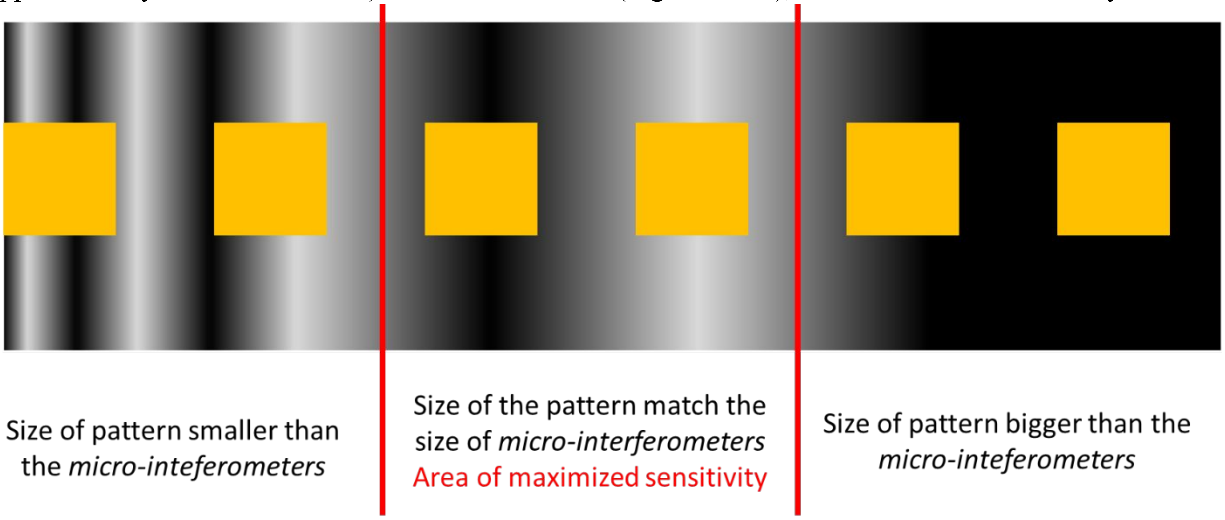

Figure S2: Scheme comparing the size of the micro-interferometers with the size of the interference pattern. The yellow squares represent the micro interferometers of 30μm side. The black and white waves represent the interference pattern. Where the size of the local interference wave matches the micro-interferometers size the sensitivity of the device is expected to be maximized.

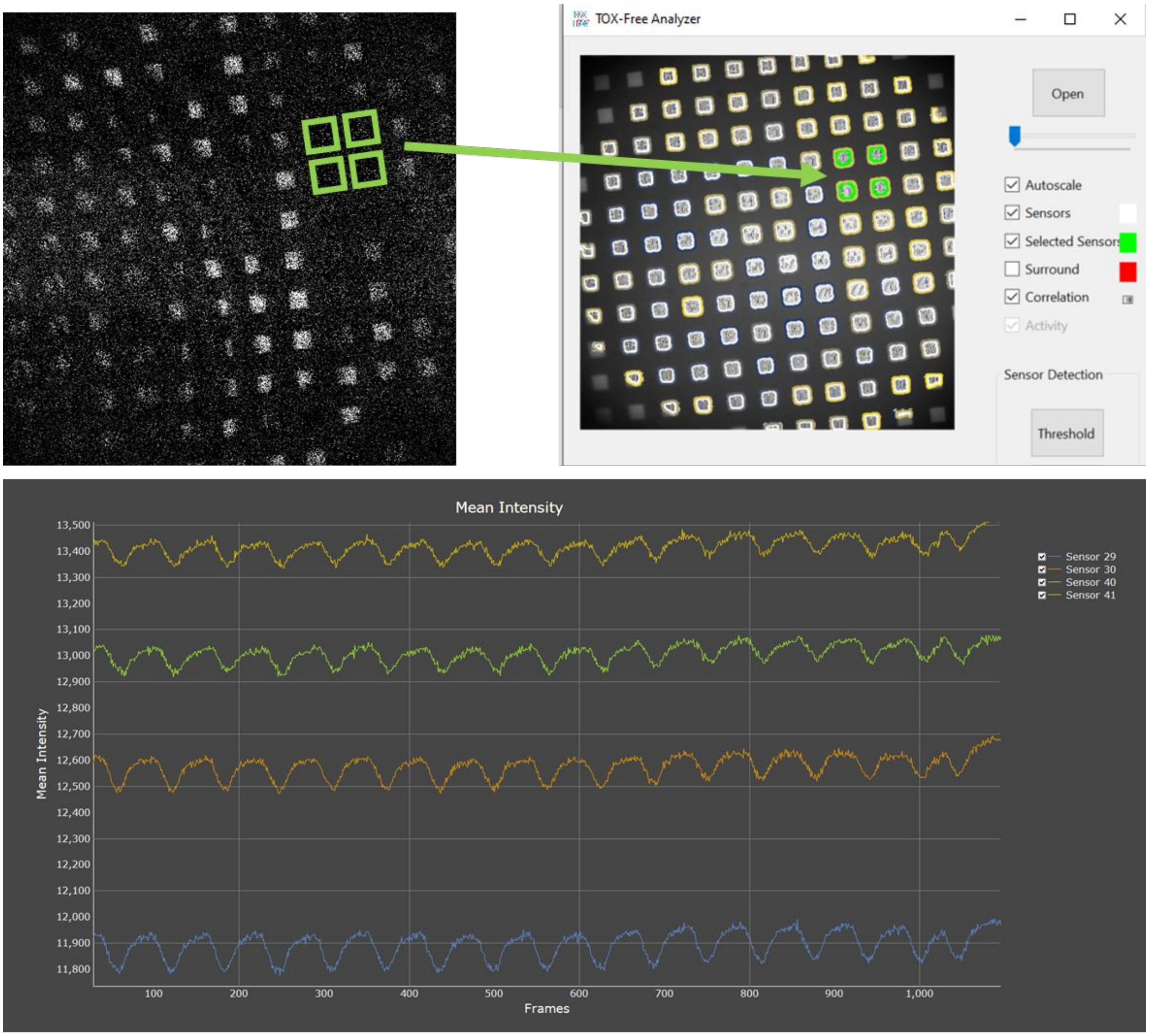

Figure S3: Scheme describing the post-processing of the data. a) Fluorescence image after subtracting the first frame from the video. The bottom-left part of the image show interference pattern that is locally smaller than the micro-interferometers. The top-right show interference pattern which size match the micro-interferometer. b) Tox-free software interface showing the same recording where we select the same sensors we indicated in the previous step. c) Fluorescence raw data traces extracted from the 4 selected sensors.

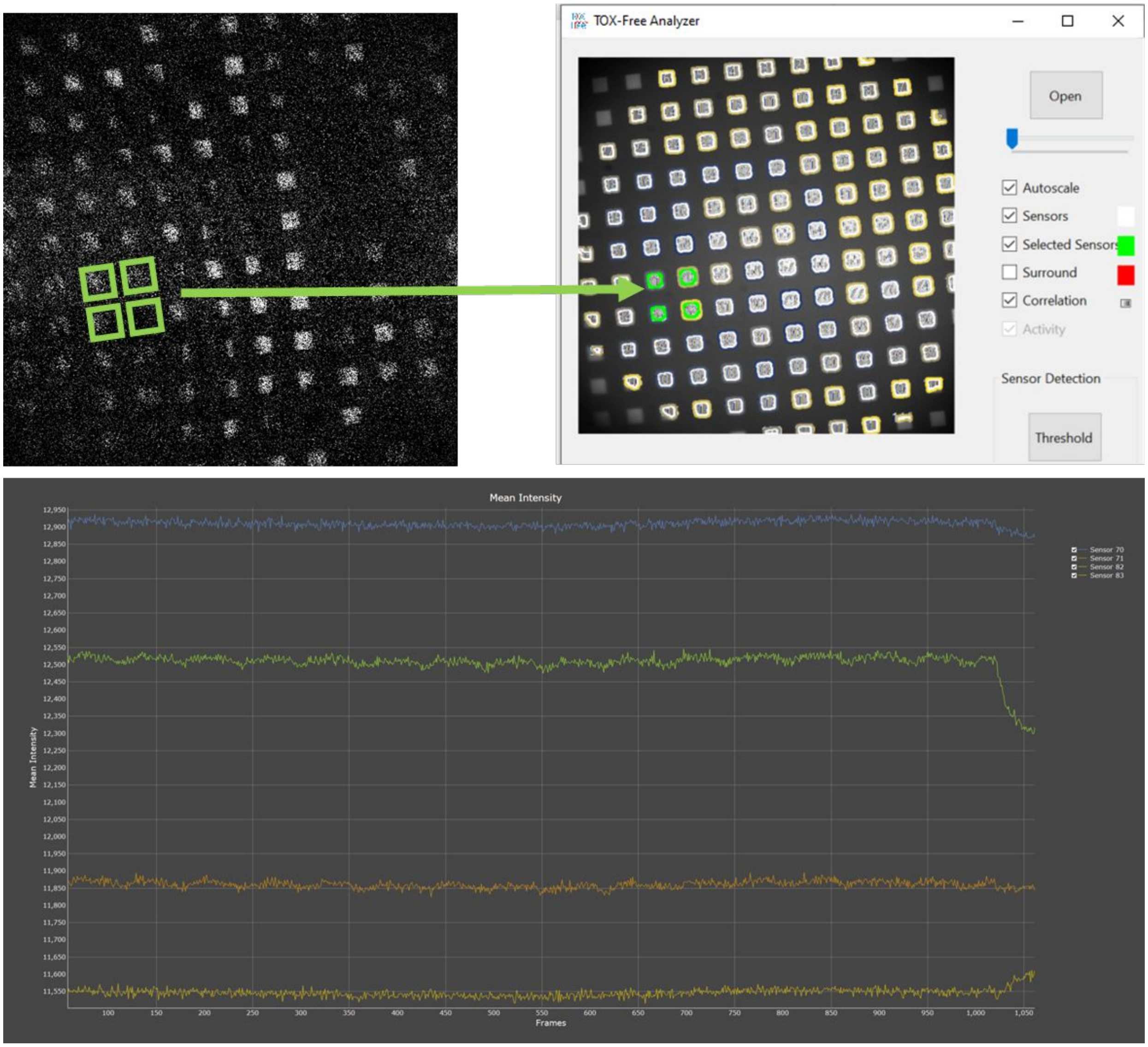

Figure S4: Scheme describing the post-processing of the data. a) Fluorescence image after subtracting the first frame of the video recording. The bottom-left part of the image show interference pattern that is locally smaller than the micro-interferometers. The top-right show interference pattern which size match the micro-interferometer. b) Tox-free software interface showing the same recording where we select the same pattern we indicated in the previous step. c) Fluorescence raw data traces extracted from the 4 selected pads.

3 Calcium Imaging

In cardiac tissue, cells synchronize their beating rhythmically, causing deformation of the entire membrane that mirrors the tissue's motion. This enables us to assess collective or overall behavior locally, thereby improving measurement quality by allowing simultaneous measurements at multiple points (N) and enhancing statistical significance and measurement precision. This approach may lead to the local detection of substrate deformation due to cardiomyocyte contraction, which depends on the oscillation modes of the thin membrane. Moreover, partial adhesion of one cell on a single gold pad will not affect the measurement, as deformation is a direct consequence of the global contraction of the monolayer. Thanks to the gold pads, most of the substrate surface remains transparent. The device's transparent areas allows for brightfield inverted imaging of the cell culture (Figure S5a) during cell seeding, facilitating easy observation and monitoring of cell condition throughout maturation. Additionally, it enhances compatibility with other optical techniques, such as calcium imaging (Figure S5b-d). At day 12 post-plating, hiPSC-CMs cultured on the devices were incubated with 2.5 μM Fluo-4 AM (Invitrogen) for 40 minutes at 37°C. Excess dye was removed by washing the cells with culture medium. Fluo-4 AM dye was excited at a 490 nm wavelength and the emission was recorded through an Alexa 488 band-pass filter cube. See video 2.

Figure S5 reports a summary of the applications allowed thanks to the transparent characteristic of the device. Preliminary measurements (Figure S5e) demonstrate the compatibility of the device with classical optical techniques for electrophysiological recording such as calcium imaging acquisition and confocal applications. We report for completeness in Figure S5c an image of the immunostaining: cardiac troponin T (green signal) is expressed as expected for a mature culture of hiPSC-CMs; NKX2-5 (red signal) marks of cardiac progenitor cells.

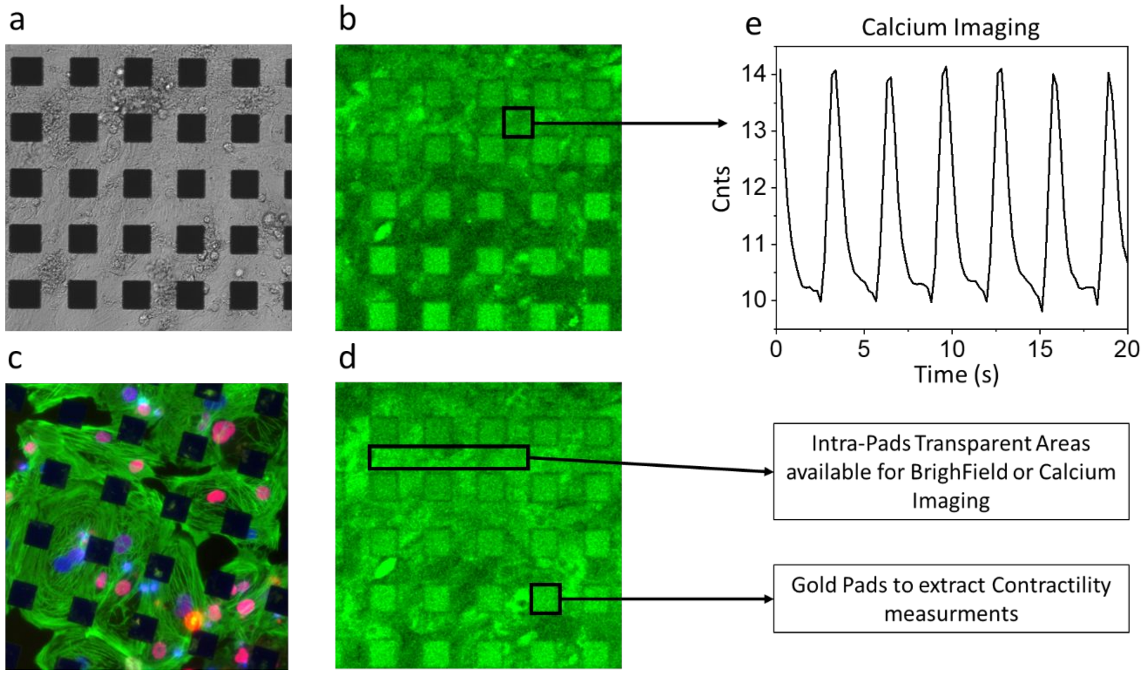

Figure S5: Multiple acquisition configuration. a) Brightfield confocal image showing cell monolayer at the inverted microscope b) Confocal calcium imaging acquisition during cell contraction. The electrical activity is propagating through the cell culture from the top to the bottom. c) Immunofluorescence d)

Confocal calcium imaging acquisition at the maximum cell contraction. e) Graph of the calcium imaging signal integrated in the area indicated by the black square in b.

4 Methods

4.1 Device fabrication

The silicon nitride membrane, 500nm thick, was fabricated from a double-sided silicon nitride-coated wafer, as previously described by Barbaglia *et.al.*[10]. Each single membrane has an area of 2x2 mm^2 and is placed in a silicon contour of 2x2 cm^2 (Figure S6b). Then, after coating the membrane with 5 nm of titanium and 20 nm of gold, deposited by e-beam evaporation (Kurt J. Lesker PVD 75), the sample underwent electrochemical gold deposition (Figure S6a 2). The deposition was performed using a plating bath containing K[Au(CN)2] dissolved in water (Parador HS – [Au] = 3 g L−1, pH = 4.2). The sample was connected to a current generator alongside a counter electrode, with the sample at the negative terminal and the counter electrode at the positive terminal. The electrodeposition process was carried out by setting the current to 35 mA for a duration of 6 minutes. Throughout the process, the plating solution was maintained at a temperature of 38°C and stirred at a moderate speed.

Subsequently, two consecutive lithography processes were carried out (Figure S6a 3-6). Firstly, a primer solution (H.M.D.S. TECHNIC) and an image-reversal photoresist (MicroChemicals AZ5214) were spun at 4000rpm for 1 minute and baked at 112 °C for 1 minute. A mask was designed with a matrix of empty square-shaped structures of 30µm in size, and exposure was performed. Afterward, the photoresist was inverted through a post-exposure bake at 120 °C for 2 minutes, followed by a flood UV light exposure of 30 seconds. After developing in AZ5214 Developer (MicroChemicals) for 45 seconds, gold etching for 30 seconds (Merck, gold etchant standard) was performed.

Once the first pattern was completed, it was used as a mask for the second lithography on the hollow part of the membrane. This side of the device is not suitable for standard lithography since it is not flat. Therefore, Lor3B was spun (1 minute, 3000rpm) and baked (3 minutes, 180 °C) on the hollow side of the device. Subsequently, an image-reversal photoresist (MicroChemicals AZ5214) was spun at 4000 rpm for 60 seconds and then baked at 112 °C for 1 minute. The sample was exposed without a mask under UV light for 10 seconds with the flat side upward. This process mirrored the pattern from one side of the membrane to the other (Figure S6a5). After following the photoresist protocol, it was inverted and developed as described above. Then, the sample was coated with 5nm Ti and 20nm Au by e-beam evaporation from the hollow side. Afterwards, 50nm of Ag/Au alloy was sputtered onto it. The surplus material was then removed by lift-off in MF319 (Figure S6a6). The silver was de-alloyed by immersing the sample in a 1:1 solution of HNO3 for 30 minutes. Finally, the sample was coated with a 3nm layer of SiO2 by ALD (Atomic Layer Deposition). The well for cell culturing was created by attaching a glass ring with a 10mm diameter using a not-cured poly(dimethylsiloxane) (PDMS) preparation (Sylgard 184) baked at 70˚C for 2 hours (Figure S6b).

The bottom mirror was created from a squared 2 cm side substrate made of SiO2 coated with 100nm of ITO by RF Argon sputtering. Finally, a layer of 5nm Ti plus 20nm Au was deposited on the ITO film by e-beam evaporation. Then, 3nm of SiO2 was deposited by ALD. Before measuring, the bottom mirror was treated with oxygen plasma (time = 300 seconds, power = 100 W, O2 gas pressure = 100%) to enhance the surface wettability.

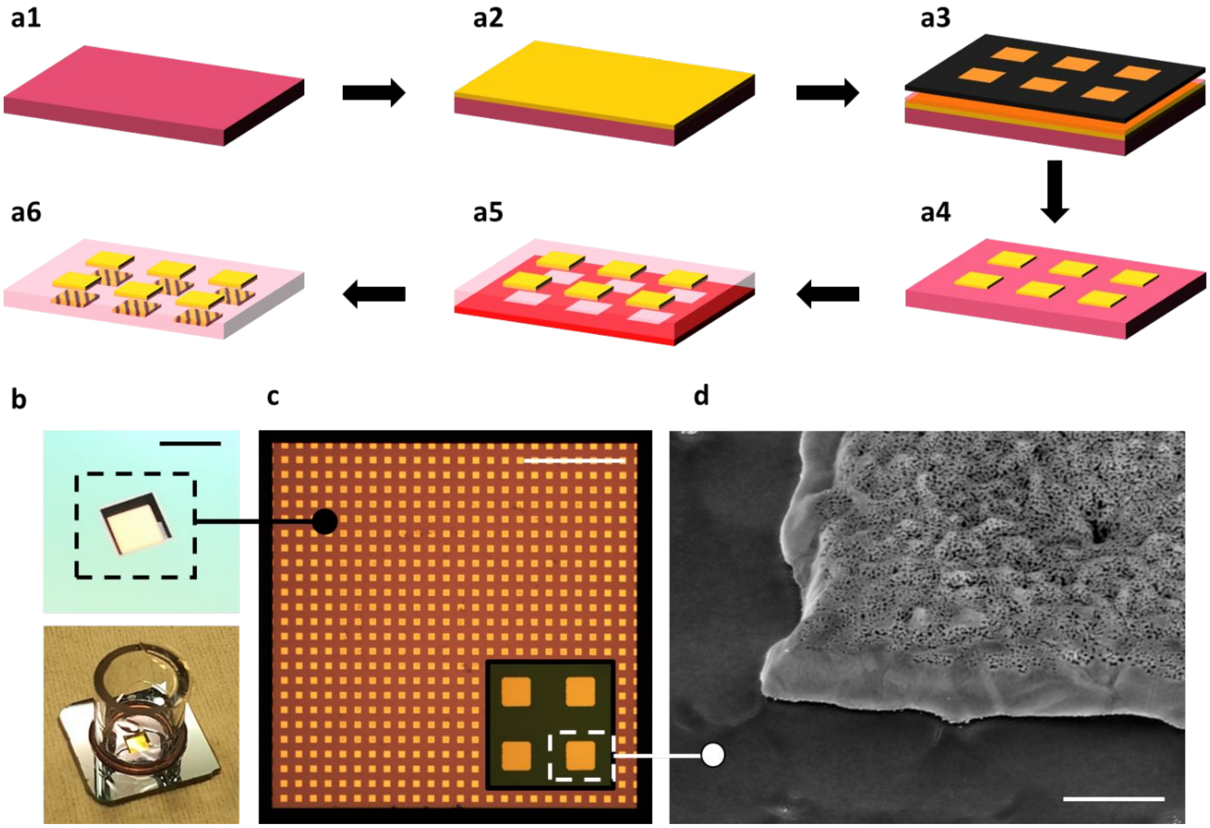

Figure S6: Device fabrication: (a) lithography process that allows to transfer the top pattern to the bottom side of the membrane. (a2) The silicon nitride membrane is covered with a thin layer of gold; (a3-a4) Optical lithography, followed by a wet etching process, allow to obtain a matrix of squared gold mirror; (a5) the sample is coated with photoresist on the bottom side, and the matrix of gold squares is used as mask for the photoresist; (a6) After developing, deposition of adhesion layer followed by the a silver-gold alloy is deposited on the bottom side. The mirrored pattern is obtained by lift-off process. Thereafter, the silver is de-alloyed in a nitric acid solution, leading to a porous gold surface. (b) Image of the device from the trans-chamber and detail of the silicon nitride membrane (scale bar: 2.5mm). (c) 5x optical microscope image of the gold pattern micro sensor (scale bar: 400μm). (d) SEM image of the porous gold layer deposited on each gold pads (scale bar: 100nm).

4.2 Experimental setup

The experimental setup employed in this study comprises an inverted microscope (Nikon Eclipse Ti2) equipped with a TRITC fluorescence filter cube set (Nikon: excitation wavelength λex = 543/22 nm, emission wavelength λem = 591.5/43 nm). We utilized a LED light source (pE-300lite, CoolLed) for fluorescence excitation. The system employed long working distance objectives (Nikon 20×/0.45 and Nikon 60×/0.70) to focus and collect both exciting light and fluorescence. To detect the output fluorescence, we integrated a CMOS camera (Photometrics-Kinetics) into the setup, which featured 10.24 megapixel resolution, the ability to bin up to 4×, and high sensitivity and speed capabilities (up to 498 frames per second across the entire field of view fullframe). A computer equipped with Micro Manager 2.0 software controlled the CMOS camera. Additionally, we incorporated a halogen lamp above the microscope to facilitate sample observation in transmission mode.

4.3 Cell culture

Human induced pluripotent stem cell-derived cardiomyocytes (hiPS-CM; iCell cardiomyocytes) were purchased from FUJIFILM Cellular Dynamics, Inc. (FCDI). The devices were previously sterilized by ultraviolet exposure for 30 min and then coated with 50 µg/ml fibronectin (Roche Applied Science) for 1 h at 37°C, 5% CO_2 in a humidified incubator. iCell cardiomyocytes were directly seeded on the devices at a density of 10,000 cells per device and grown according to the specifications of the commercial supplier. Recording of cardiomyocytes contraction were performed starting from 8 days post-plating.

4.4 Data analysis

The data obtained in this study comprised video recordings in .tif format.The .tif have been analyzed by Tox-FREE software (https://toxfreeproject.eu/results). The results obtained from the software were saved in .csv format and subsequently imported into OriginPro 2020a (OriginLab) for further data analysis.

5 References

(1) Ventsel, E.; Krauthammer, T. *Thin Plates and Shells : Theory, Analysis, and Applications*; Marcel Dekker New York: New York SE -, 2001. https://doi.org/LK - https://worldcat.org/title/47168757.

(2) Maddux, G. E.; (U.S.), A. F. F. D. L. *Stress Analysis Manual*; Air Force Flight Dynamics Laboratory, Air Force Systems Command Wright-Patterson Air Force Base, Ohio: Wright-Patterson Air Force Base, Ohio SE -, 1970. https://doi.org/LK - https://worldcat.org/title/12959772.

(3) İmrak, C.; Gerdemeli, İ. An Exact Solution for the Deflection of a Clamped Rectangular Plate under Uniform Load. *Appl. Math. Sci.* **2007**, *1*.

(4) Hakim, G.; Abramovich, H. Large Deflections of Thin-Walled Plates under Transverse Loading-Investigation of the Generated In-Plane Stresses. *Mater. (Basel, Switzerland)* **2022**, *15* (4). https://doi.org/10.3390/ma15041577.

(5) Maddux, G. E.; Vorst, L. A.; Giessler, F.; Moritz, T. Stress Analysis Manual; 1969.

(6) Campbell, D. P. Interferometric Biosensors BT - Principles of Bacterial Detection: Biosensors, Recognition Receptors and Microsystems; Zourob, M., Elwary, S., Turner, A., Eds.; Springer New

York: New York, NY, 2008; pp 169–211. https://doi.org/10.1007/978-0-387-75113-9_9.
